# Driving Behaviour in Depression Based on Subjective Evaluation and Data from a Driving Simulator

**DOI:** 10.3390/ijerph20085609

**Published:** 2023-04-21

**Authors:** Vagioula Tsoutsi, Maria Papadakaki, George Yannis, Dimosthenis Pavlou, Maria Basta, Joannes Chliaoutakis, Dimitris Dikeos

**Affiliations:** 1 First Department of Psychiatry, Medical School, National & Kapodistrian University of Athens, Eginition Hospital, 11528 Athens, Greece; ddikeos@med.uoa.gr; 2 Laboratory of Health and Road Safety, Department of Social Work, School of Health Sciences, Hellenic Mediterranean University, 71410 Crete, Greece; mpapadakaki@hmu.gr (M.P.); jchlia@hmu.gr (J.C.); 3 Department of Transportation Planning and Engineering, School of Civil Engineering, National Technical University of Athens, 15773 Athens, Greece; geyannis@central.ntua.gr (G.Y.); dpavlou@scientra.gr (D.P.); 4 Department of Psychiatry, University Hospital of Heraklion, 71500 Crete, Greece; mbasta73@gmail.com

**Keywords:** depression, sleep disorders, driving behaviour, driving simulator

## Abstract

Road traffic collisions are a major issue for public health. Depression is characterized by mental, emotional and executive dysfunction, which may have an impact on driving behaviour. Patients with depression (N = 39) and healthy controls (N = 30) were asked to complete questionnaires and to drive on a driving simulator in different scenarios. Driving simulator data included speed, safety distance from the preceding vehicle and lateral position. Demographic and medical information, insomnia (Athens Insomnia Scale, AIS), sleepiness (Epworth Sleepiness Scale, ESS), fatigue (Fatigue Severity Scale, FSS), symptoms of sleep apnoea (StopBang Questionnaire) and driving (Driver Stress Inventory, DSI and Driver Behaviour Questionnaire, DBQ) were assessed. Gender and age influenced almost all variables. The group of patients with depression did not differ from controls regarding driving behaviour as assessed through questionnaires; on the driving simulator, patients kept a longer safety distance. Subjective fatigue was positively associated with aggression, dislike of driving, hazard monitoring and violations as assessed by questionnaires. ESS and AIS scores were positively associated with keeping a longer safety distance and with Lateral Position Standard Deviation (LPSD), denoting lower ability to keep a stable position. It seems that, although certain symptoms of depression (insomnia, fatigue and somnolence) may affect driving performance, patients drive more carefully eliminating, thus, their impact.

## 1. Introduction

Road traffic collisions (RTCs) and the related casualties are one of the public health challenges that threaten people’s health and impose huge costs for health systems and society. In most countries RTCs cost 3% of their gross domestic product [1]. According to the World Health Organization (WHO) predictions, RTCs will increase by 28% by the year 2030 and will become the fifth leading cause of death [2]. Nowadays RTCs are the eighth highest cause of death worldwide; about 1.35 million people die each year due to RTCs, while 20 to 50 million injuries per year are due to them. They also seem to be the leading cause of death for children and young adults aged 5–29 years [1]. According to the WHO relevant observatory a user of the road network loses their life every 23 s [3].

The human factor is considered to outweigh any other factor leading to RTCs, such as vehicle mechanical error or road structural faults and maintenance failure. According to research, the human factor has a direct effect on 93% of crashes, and this makes human behaviour the main cause of RTCs [4]. Thus, the majority of studies on RTCs have focused mainly on the driver and only a small number have examined the effect of mental disorders on driving performance; the majority of these studies have focused on the effects of drugs, especially antidepressants and benzodiazepines [5,6,7,8]. The ways to examine driving performance include the use of questionnaires, various neuropsychological tests (such as brief mental status examinations, visual perception and visual spatial abilities, attention, processing speed, language, memory, executive functioning, awareness and metacognition) [9] and driving under “natural” conditions (on especially equipped cars, fitted with cameras and other instruments for the assessment of driving behaviour) or on a driving simulator. In a driving simulator a virtual road environment resembling driving on the road under realistic conditions is generated by computer programming tools, which allow for the “creation” of various driving scenarios and road conditions, such as different traffic loads, driving day or night, different weather and road conditions, presence or not of unexpected events, etc. [10]. Moreover, driving simulators are much cheaper and more accessible to larger number of people than the specially equipped cars and have many different uses, such as for educational purposes, detection of fitness to drive especially among professional drivers and research into the areas of driving safety and/or driving under substances and/or alcohol use or other situations [10]. According to a systematic review, the use of driving simulators in medical research has increased; in PubMed there were only 32 relevant published articles published in 1999 while in 2015 there were 327 articles [10]. It seems that real driving and driving simulation are comparable for measuring driving behaviour, although there are some aspects that may be better assessed by using a driving simulator (e.g., reaction time, effects of self-evaluated sleepiness, line crossings) [11]. For all these reasons driving simulators are considered to be a preferable means for the assessment of driving ability. 

The WHO claim that mental and behavioural disorders keep on increasing and that in 2030 depression will be the first cause of disability [12,13]. The prevalence of depression in the general population seems to be 4.7% (4.4–5.0%) [14,15]. Depression is characterised by mental, emotional and executive dysfunction; cognitive and psychomotor functions are often being affected, more in the elderly than in younger patients [16,17]. Sleep disorders are very common in depression, either insomnia or hypersomnia [18]. Symptoms of depression or side effects of its treatment, such as somnolence, are expected to affect a person’s daily life as well as their functioning level with the potential of also influencing driving ability [19,20,21], while insomnia has also been considered as an independent risk factor for RTCs [22,23].

The relationship between depression and driving has been examined in a number of studies [5,6,7,19,20,21,24,25,26,27,28,29]. A higher risk of RTCs has been found among depressive patients as compared to control subjects not suffering from depression in some studies of forensic examination of already recorded RTCs [7,19,21], although other similar studies have concluded against such an association [27,29]. The driving ability of depressed patients has been examined in a few studies on a driving simulator [20,25,26,28], and on other studies on the road using a specially fitted car [5,24]. Some of these studies have found an association between depression and decline in driving ability [5,20,24,25], while others have not [27,28,29]. It is worth noticing that of those studies which show that patients with depression have a somewhat compromised driving ability, some that report that this effect is mitigated by treatment with antidepressants [5,25]. In a preliminary report, in which 13 patients with depression and 18 healthy controls were included, we found certain driving attitude characteristics and specific driving simulator measurements (lateral position, velocity, distance from the preceding vehicle) to be related with depression and sleep disturbance [28]. 

The aim of the present study was to confirm and further expand previous findings on the effects of depression and its symptoms on driving performance based on a larger sample, by employing an innovative approach using triangulation in exploring the effects of depression on driving performance via subjective and objective measures. Sleep disorders, either as a symptom of depression or as a side effect of its treatment, were particularly focused on. Studying driving in depression is important because no uniform guidelines exist regarding fitness to drive and licensure for patients with depression [30], while there is a lack of knowledge among health professionals on how to deal with depression and driving [31,32] and, usually, the process relies on patients’ self-awareness of their driving difficulties due to illness and self-reporting to licensing authorities.

The structure of this paper is as follows: The Methods section contains the description of the study design and sample, the presentation of the questionnaires, the description of the driving simulators which have been used, and the statistical analysis methods employed. In the Results section, after the description of main characteristics of the sample, the results pertaining to the driving questionnaires are presented, followed by the results derived from the driving simulators. In a similar format, the Discussion first refers to the driving questionnaires, and then to the driving simulator findings. The paper closes with an overall conclusion.

## 2. Methods

### 2.1. Study Design and Sample

Driving behaviour and attitude towards driving were assessed in a group of patients with depression and a group of healthy controls. The study had two parts: In the first part of the study, participants were asked to complete scales and questionnaires regarding demographics (gender, age, marital status) and medical history (height, weight, presence of medical disease, use of alcohol/drugs, and for patients with depression age of onset, family history, number of episodes/hospitalisations, medication or other types of therapy), scales on symptoms of depression and sleep disorders and questionnaires on driving attitude and behaviour. In the second part, participants were asked to drive on a driving simulator in a motorway (MW) scenario, and under low (L) and high (H) traffic conditions in urban (U) and rural (R) scenarios, all in good weather conditions.

The sample of the present study consisted of patients from outpatient psychiatric services diagnosed with depression. The First Department of Psychiatry of the National and Kapodistrian University of Athens (NKUA), Eginition Hospital, Athens, Greece, the Department of Psychiatry of the University Hospital of Heraklion, Crete, Greece and other collaborating private centres were included following the approval of the project by the institutional ethics committees. A total sample of 39 depressed patients, 12 of whom were not receiving antidepressant medication, and 30 healthy controls participated in the study. Inclusion criteria for all participants were to have a driver’s license and to have driven on a regular basis during the last six months. Exclusion criteria were the presence of either neurological problems or severe vision or hearing loss. All patients were clinically diagnosed based on the current diagnostic criteria (DSM-5) [33] and were followed by an experienced psychiatrist. Patients were informed on the study by their treating psychiatrist, then the procedure was fully explained by the researcher and, finally, after their written consent, a relevant appointment was set to participate in the procedure. Regarding participants in the control group, a volunteer sample was recruited via a public invitation through the university networks and upon controlling for gender, age and years of driving experience as matching variables with the intervention group. The same process of explanation and consent was followed in the control group. 

### 2.2. Questionnaires and Scales

The following questionnaires and scales were used.

#### 2.2.1. Demographics and Medical History

An improvised questionnaire was used to collect information on age, gender, marital status, mental and somatic disorders, current illness(es), medication, family history and current mental status. Height and body weight were measured; body mass index (BMI) was calculated. 

#### 2.2.2. Beck Depression Inventory (BDI)

The BDI is a multiple-choice self-report inventory of 21 items, one of the most widely used psychometric tools for assessing symptoms of depression. Each item has a set of four possible responses, ranging in intensity from 0 to 3. Higher scores indicate higher severity of depressive symptoms [34].

#### 2.2.3. Athens Insomnia Scale

The Athens Insomnia Scale is a widely used self-administered instrument and was developed to assess sleep difficulty based on the ICD-10 criteria [35]. It consists of 8 items to be scored on a four-point Likert scale; of these items the first 5 are related to assessing any difficulty with sleep induction, awakenings during the night, early morning awakening, total sleep time and overall quality of sleep, while the last 3 items are related to problems with sense of well-being, functioning and sleepiness during the next day. Total score ranges from 0 to 24; a score of 6 or higher is considered to denote the presence of insomnia [36].

#### 2.2.4. Epworth Sleepiness Scale (ESS)

The ESS scale is a self-reported scale consisting of 8 questions; respondents are asked to rate on a four-point Likert scale the likelihood of falling asleep while engaging in eight different activities [37]. Most people engage in these activities almost every day. The scale ranges from 0 to 24. The higher the ESS score, the higher the tendency for the responder to fall asleep or to feel drowsy during the day.

#### 2.2.5. Fatigue Severity Scale (FSS)

The FSS is a self-reported scale consisting of 9 items evaluating the severity of symptoms of fatigue, with respondents asked to score each one of them from 1 to 7, where 1 means “strongly disagree” and 7 “strongly agree” [38].

#### 2.2.6. StopBang

The StopBang questionnaire consists of 8 items evaluating the risk of possible sleep apnoea [39]; the respondents are asked to check each item as “yes” or “no”. From 0 to 2 positive answers means that there is a low risk for the presence of obstructive sleep apnoea, from 3 to 4 a moderate risk and from 5 to 8 a high risk. 

#### 2.2.7. Driver Stress Inventory—DSI

The DSI aims to detect driver reactions when driving under stress and was developed by Matthews et al. in 1996 [40], as an extension of Driving Behavior Inventory (DBI) by Glendon et al. [41]. It is a self-reported questionnaire which assesses propensity to develop stress reactions while driving. It includes 48 items and contains five subscales for driving aggression, dislike of driving, hazard monitoring, thrill seeking and proneness to fatigue. The respondents indicate how strongly they agree with each item on an 11-point Likert scale that ranges from 0 (“not at all”) to 10 (“very much”). 

#### 2.2.8. Driver Behaviour Questionnaire—DBQ

The DBQ is a self-reported questionnaire; it consists of 26 items and produces scores of three subscales: driving errors, traffic violations and attention lapses [42]. The participants are asked to respond on a five-point Likert scale from 1 (“never”) to 5 (“always”) according to how frequently they had committed each of the 26 behaviours. 

### 2.3. Driving Simulation

In the current study two different driving simulators were used: the first, “Virage VS500M”, set at the Laboratory of Health and Road Safety (LaHeRS), Department of Social Work, Hellenic Mediterranean University (HMU) in Heraklion, Crete and the second, “Foerst Driving Simulator FPF F10P”, set at the Department of Transportation Planning and Engineering, School of Civil Engineering, National Technical University (NTU) of Athens. 

Both driving simulators consist of an open cabin with motion systems including an adjustable driver’s seat, the central console (steering wheel, 5-gear lever plus rear, pedals, accelerator, brake, clutch), dashboard (speedometer, tachometer), light switch, warning lights, horn, turn signals, wipers and starter. The driving environment is equipped with real car parts to provide a realistic sense, which is supported by the appropriate sounds and vibrations. Graphics are displayed on three wide screens that cover an approximately 180-degree field of view and are in front of the cabin. The mirrors are embedded in the screens. 

In this study, different scenarios were employed according to the study objectives. In LaHeRS, HMU Virage Simulator, participants were asked to drive in two different simulator scenarios; on a motorway and in the urban environment (i.e., in the city), under normal (low) traffic conditions. In the NTU Foerst Driving Simulator the participants were asked to drive in five different simulation scenarios: in an urban environment (under low and under high traffic), in a rural road (under low and under high traffic) and on the motorway under normal (low) traffic conditions.

The three main driving performance domains which were analysed were average speed, average safety margin from the preceding vehicle and standard deviation of lateral position (denoting difficulty in maintaining a steady driving path). Speed was measured in kilometres per hour. Safety margin was assessed by two variables: HWay (distance in m from the vehicle driving ahead) and THead (time in seconds to “headway”, i.e., to collision with the vehicle driving ahead). Lateral position was assessed by RSpur (distance in metres from the middle of the road in m).

### 2.4. Statistical Analysis

Data are presented as means ± SD or as percentages. All variables were checked for normality by the Shapiro–Wilk test. Initially, chi-square, Student’s *t*-test or Mann–Whitney test was used, as appropriate, to assess differences between groups of patients and controls. 

Backward Multiple Linear Regression (BMLR) analyses were then performed, with the aim to identify which of the independent variables influence the driving aspects (dependent variables), i.e., those derived from the questionnaires as well as those derived from the driving simulator. The method of BMLR was chosen following the suggestion that, while regression models often produce weaker predictions when used for large data samples, they generally work very well for a small sample of data [43], as well as the suggestion that, by starting with the full model, backward regression has the advantage of considering the effects of all variables simultaneously, something which is especially important in case of variables in a model being correlated which each other [44], as is the case with some of our independent variables (AIS with FSS, StopBang with FSS and BMI).

The independent variables for the BMLR were gender, age, group (patient/control), research centre (Athens/Heraklion), BMI and total scores of ESS, FSS, AIS and StopBang. The dependent variables were the total score of the 5 subscales (aggression, dislike of driving, hazard monitoring, thrill seeking and proneness to fatigue) of the DSI, the total score of the 3 subscales (driving errors, traffic violations and attention lapses) of the DBQ, and the driving simulator variables of the three driving performance domains, i.e., average speed, average safety margin (HWay, THead) and standard deviation of lateral position (RSpur), each in the various road scenarios (MW, motorway; UL, urban environment—low traffic; UH, urban environment—high traffic; RL, rural environment—low traffic; RH, urban environment—high traffic). Since each domain consisted of more than one variable, Bonferroni correction for multiple comparisons was applied taking into the number of variables per domain (five for average speed, ten for average safety margin and four for lateral position standard deviation). 

The level of statistical significance was set at 5%. Given the means and standard deviations of our variables, power analysis showed that there was about 80% power to detect a difference of about 15–20% between groups for our population size.

The SPSS 23.0 (IBM SPSS Statistics for Windows, Version 23.0. Armonk, NY, USA: IBM Corp., 2015) statistical package was used for the analysis. 

## 3. Results

### 3.1. Main Characteristics of the Sample 

Most of our sample were women (65.2% of the total sample, 66.7% of patients, 63.3% of controls) and about half of the total sample were married or cohabited (52.2% of the total sample, 53.8% of patients, 50.0% controls). The mean age was 45.22 ± 11.34 years old for the total sample (range 24 to 74); 46.97 ± 12.29 for patients (range 24 to 74) and 42.93 ± 9.71 for controls (range 27 to 67). Respective values for BMI were 25.67 ± 5.19, 25.88 ± 6.28 and 25.39 ± 3.35. No significant differences were detected between patients and controls for the above variables. Mean score of depressive symptoms as assessed by the BDI was significantly higher in patients compared to control subjects (17.56 ± 9.17 vs. 5.66 ± 4.14). Patients had significantly higher scores than the controls on the scales for fatigue (FSS; 37.44 ± 14.46 vs. 29.17 ± 9.58), insomnia (AIS; 8.64 ± 5.25 vs. 4.77 ± 3.58) and sleep apnoea (StopBang; 2.23 ± 1.30 vs. 1.10 ± 1.12); the two groups did not differ in sleepiness (ESS; 6.97 ± 4.27 vs. 6.53 ± 3.42).

### 3.2. Driving Questionnaires (DSI and DBQ) 

Scores in the driving questionnaires did not differ between patients and controls (Table 1 and Table 2). In the linear regression analysis of DSI and DBQ subscales scores, it was found that aggression and dislike of driving were associated with fatigue; thrill seeking and proneness to fatigue with male gender; traffic violations with younger age and fatigue; driving errors with lower BMI; and hazard monitoring with male gender, lower BMI and fatigue (Table 2). 

### 3.3. Driving Simulator

Table 3 presents the final models of the backward linear regression of the driving simulator variables from both groups (patient/control), demographic characteristics, BMI and scores of scales for sleepiness (ESS), fatigue (FSS), insomnia (AIS) and probability for sleep apnoea (StopBang). Simulator variables for the whole sample of both sites (Heraklion and Athens, N = 69) were available only in the motorway and urban low traffic environments. Results for other environments (urban high traffic, rural low and high traffic) derive only from the Athens sample (N = 38). Speed and lateral position were assessed by a single measure each, but for safety margin there were two variables.

As shown in Table 3, speed was found to be influenced only by gender and age: average speed was lower in women than in men by 7.95 km/h for the motorway environment (corresponding to 11% of the average speed of the whole sample, which was around 68 km/h), 2.23 to 2.31 km/h for the urban environment (about 10% of the average speed) and 3.99 to 5.89 km/h for the rural environment (about 13% of the average speed); it declined per decade of age by 4.85 km/h for the motorway and from 1 to 3 km/h for the other environments. Regarding safety margin from the preceding vehicle, it was found to be higher with advancing age in practically all driving environments (measured as either distance from the preceding vehicle or as time to collision), for women in the rural high traffic environment (about 11 s longer time to collision than men), and it was also positively correlated with somnolence (for urban environment), insomnia (on the motorway) and symptoms of sleep apnoea (on the motorway and urban low traffic environments, but the effect was reversed for rural low traffic environment); safety margin was negatively correlated with BMI (motorway and urban low traffic environments). Depression was found to be associated with a higher safety margin, measured as time to collision, being 16.3 s higher in patients than in controls, but only in the rural low traffic environment. Finally, lateral position stability was compromised (higher standard deviation values) for people with a higher AIS score for insomnia (urban high traffic condition), higher ESS score (on the motorway), as well as with the advancement of age (motorway) and women drivers (urban low traffic condition). Lateral stability seemed to be higher for patients with depression versus controls in the urban high traffic condition and worse in the rural high traffic condition.

## 4. Discussion

The current study evaluated the driving performance in patients with depression and healthy controls by using relevant questionnaires and testing on a driving simulator. 

### 4.1. Questionnaire Findings

The group of patients with depression did not differ from controls regarding stress of driving and driving behaviour as assessed through the questionnaires DSI and DBQ, respectively. 

There have been a limited number of studies using the DBQ and the DSI (or other versions of these questionnaires) on patients with psychiatric disorders (including depression, but not always separately examined). In these studies, some specific items of the questionnaires are reported to show weak to moderate correlations with certain symptoms, but not with depression per se [45,46,47,48,49,50]. Thus, the present study is the first comparison between patients with depression and controls regarding DSI and DBQ and it shows that there is no difference whatsoever between patients with depression on these questionnaires assessing stress of driving and driving behaviour. 

The subjective feeling of fatigue (which is often a symptom of depression), as assessed by FSS, was found to be positively correlated to driving aggression, dislike of driving, impaired hazard monitoring and reported road traffic violations. Aggressive driving has been identified as an important factor related to RTCs [51], and various attempts to explain driver emotions and decision-making are being currently pursued [52]. Studies from the literature show that the presence of fatigue impairs various aspects of driving ability [53] and increases the risk of causing a serious road traffic accident [54]. The effects of fatigue are comparable to those after alcohol intake [55], while driver incapacitation due to drowsiness and fatigue is one of the major causes resulting in fatal traffic collisions [56]. In our study fatigue is shown to cause extra nervousness and aggression, which are likely to increase unpleasant feelings and dislike to driving, but the relationship with RTCs could not be confirmed. This is probably due to the assessment through self-reports and to the small number of our sample, in comparison to the above and other relevant studies, which refer to greater samples usually based on already recorded accidents. Nevertheless, fatigue generated by extensive driving has a limited impact on driving skills if drivers take breaks to rest, even if these are short [57]. 

Other parameters, such as gender, age and, to a lesser extent, body mass index, were found to affect driver stress and behaviour. Women showed lower hazard monitoring, less thrill seeking through driving and less proneness to fatigue while driving. Thrill seeking through driving has been found to be related to male gender, also in another study [58]. Older age was found to be associated with fewer reported traffic violations. This finding is consistent with the literature, which shows that older drivers are less likely to be involved in RTCs, while younger drivers are more likely to be fatally injured in an RTC, with the adjusted crash risk peaking at age 20–29 years and decreasing gradually at age 60–69 years [7,59,60,61,62]. Finally, BMI has been previously reported as an important risk factor for causing road traffic collisions [63]. In our study, although BMI was found to be associated with fewer reported driving errors, it was also found to be associated with reduced hazard monitoring. This may be related to excessive daytime sleepiness, sleep apnoea and depression, which all have been found to be associated with obesity [64,65,66].

### 4.2. Driving Simulator Findings

Similarly to our findings, another study using a driving simulator failed to find difference in average speed values between patients with depression (N = 18) and controls (N = 29) [20]; our study confirms these findings in a larger sample of patients with depression (N = 39). Safety margin from the preceding vehicle was found to be higher for patients with depression in the rural scenario with low traffic. Regarding depression and lateral position our study produced contradictory results. While in the urban scenario patients with depression were keeping a stable path, in the rural scenario they demonstrated a greater LPSD. A case-control study found that medicated patients with depression demonstrated a greater weaving motion (greater LPSD) [24]. In another study, using an on-the-road driving test, authors report that the mean LPSD of untreated and (to a lesser degree) of treated patients with depression was significantly higher than that of controls [5].

As seen above, in our study we found no major differences between patients with depression and controls in objective measures of driving. Research so far has produced mixed results on whether depression is correlated or not with impaired driving as well as with higher collision or fatality rates [5,6,7,19,20,21,24,25,26,27,28,29]. A review has shown that patients with depression are not more prone to having more RTCs, but they do have more violations and perform more poorly on driving simulators [19]. 

Our study, though, shows that patients with depression have almost the same driving profile as controls. Other studies have shown that treated patients with depression have a better performance compared to those who are untreated [5,20,25,26]. It has been found also that antidepressant treatment increases driving safety, even with antidepressants which are considered to be sedative [25,26]. International guidelines do not suggest a blanket restriction on driving for individuals with psychiatric disorders; recommendations are made for an individualized approach, considering factors such as adherence to treatment, absence of cognitive impairment or sedating medications, sufficient periods of stability after acute episode of illness, lack of impact on daily functioning and insight as pre-requisites for safe driving [30,67].

Insomnia and sleep-related problems, such as sleepiness (symptoms frequently found among depressed patients) are considered to be important factors for RTCs [23]. Somnolence was found to be positively correlated to safety margin. The sleepier the participants felt, the longer the distance they kept from the preceding vehicle; a similar finding was observed for insomnia symptoms. Studies on daytime sleepiness (often a result of sleep apnoea) show that it is a serious risk factor for traffic accidents [68,69,70]. Nevertheless, in our study, sleepiness was found to be associated with keeping a longer safety distance from the preceding vehicle, possibly representing a compensatory behaviour. One other study has also found that sleepy driving is associated with increased headway in about 2/3 of the drivers; night-time shift was also associated with such effects [71]. Safety margin was also found to be negatively correlated to BMI in the present study. High BMI is known to increase the mortality rate of RTCs [72]. No studies were found regarding BMI and safety distance from the preceding vehicle. As regards LPSD (a measure of lateral instability), in our study the ability to keep a stable path was compromised by higher insomnia scale scores, although one previous study has not shown such differences [73]. Similarly to us, another study found that insomnia patients had a larger standard deviation of lateral position as well as more lane crossings [22]. 

Further to depression and its associated symptoms, factors which were found in our study to influence speed were mainly demographic characteristics. More specifically, female gender was found to be correlated with lower speed; age was found to be negatively correlated with speed, i.e., participants of older age were found to keep speed at lower levels. Studies have shown that speed is an important factor for road safety, since it is related to the risk of RTCs, while also affecting crash severity, resulting in higher mortality [74,75,76,77]. Other studies have also shown that male gender is associated with higher driving speed [78,79,80,81]. It is of note that, in one of those studies in a driving simulator, men were found to drive approximately 17% faster than women [80], a value that is not far away from our findings that on the motorway the difference in speed between men and women is about 11% on the average, increased to about 13% for a rural scenario. Regarding age, speeding and other dangerous driving behaviours are more common in younger people, particularly men [82,83]. Young drivers are also known to exceed speed limits more often than older drivers [77]. These observations are consistent with our findings. 

Safety margin from the preceding vehicle was found to be positively correlated with older age and with female gender (for the latter, only in one scenario). Previous studies have shown that ageing affects driving [84,85], but also that driving performance is affected to a lower degree than expected and commonly assumed, because probably older drivers use compensatory actions [86,87]. It is known that driving skills decline with age, and it is a possibility that the longer safety distance maintained by older participants of our sample reflects a compensatory measure in accordance with the above studies [86,87]. Finally, lateral position stability was found to decline with advancing age, in accordance with another study comparing older and younger drivers [88].

In conclusion, our findings that people with depression in certain occasions may keep a higher safety margin from the preceding vehicle, and that safety margin is also longer for symptoms such as somnolence, fatigue and insomnia suggest that patients may be using compensatory mechanisms and, thus, their driving remains safe, in spite of any impairments in driving performance.

## 5. Limitations

One of the main limitations of the current study is the fact that two different driving simulators were used introducing potential inter-equipment variability and bias. However, the scenarios and driving environments were chosen so that similar variables could be derived from both simulators. 

Recruiting patients with depression to be examined on a driving simulator is difficult and, thus, the number of our sample is relatively low, raising concerns about its representativeness of the general population of depressed patients. Furthermore, although all patients were diagnosed as suffering from Major Depression by expert psychiatrists, one has to keep in mind that mood swings (a person feeling depressed/happy for a while) due to recently faced trauma/feeling of joy are usually short periods and may also influence the reaction and control over self-feeling; these kinds of emotional reactions are not covered by the diagnosis of Major Depression. Nevertheless, low values of controls in the BDI, show that our control group did not have prominent feelings of depression at the time of examination, while the opposite was the case for patients.

Another limitation is that patients differed from controls regarding measures of fatigue, insomnia and sleep apnoea, which may explain, at least partly, differences in driving skills; however, this was taken into account by including these variables in the multiple linear regression analyses where the influence of each of these variables is demonstrated.

Heterogeneity of the sample of patients based on some receiving medication and others not, is another limitation of the study. Since less than one third of patients were medication-free, there was not enough power to statistically analyse differences between the group of patients who were receiving medication and those who did not. Furthermore, patients presented with different symptomatology and severity of symptoms at the time of the examination. Nevertheless, in all patients, severity of depression was assessed using scales and they were all diagnosed by a psychiatrist using standard criteria, constituting, thus, a well-defined population. Another strength of our study is that both subjective and objective variables were included. 

## 6. Conclusions

In the present study patients with depression were not found to differ from healthy controls regarding either driving questionnaires or the majority of findings derived from the driving simulator. We believe that this finding is genuine, since differences between gender and age have been readily found in accordance with expectations based on the literature. In addition, power analysis showed that there was about 80% power to detect a difference. Thus, we believe that if there was a difference between the two groups it would have been easily detected. Further to the contribution of the above finding to the existing literature, an added value of our study to the state of the art is that we observed specific symptoms of depression (insomnia, fatigue and somnolence) to somewhat affect driving performance, but also that patients seem to realize the effects that these symptoms have on their driving ability and, consequently, drive more carefully. More specifically, the diagnosis of depression was associated with longer distance (safety margin) from the preceding vehicle, as was the case for symptoms of depression such as fatigue, excessive sleepiness and insomnia. Health professionals can use this information to highlight to patients with depression the importance of recognising such symptoms and exert appropriate caution when behind the wheel.

Future studies can benefit from larger numbers of patients, which would be adequate to address heterogeneity issues related to the type of treatment and the mood state the patients are in at the time of the examination, including severity of depressive and associated symptoms.

## Figures and Tables

**Table 1:** Means and standard deviation of questionnaires regarding driving in patients and controls.

	Patients (N = 39)	Controls (N = 30)	Significance
Driver Stress Inventory (DSI)			
Aggression	4.49 ± 1.03	4.34 ± 1.059	N.S
Dislike of driving	4.73 ± 1.58	4.44 ± 1.18	N.S
Hazard monitoring	5.58 ± 1.51	5.06 ± 1.23	N.S
Thrill seeking	1.35 ± 1.29	1.42 ± 1.43	N.S
Proneness to fatigue	6.50 ± 2.26	6.92 ± 2.23	N.S
Driver Behaviour Questionnaire (DBQ)			
Violations	0.69 ± 0.44	0.76 ± 0.51	N.S
Errors	0.31 ± 0.30	0.32 ± 0.30	N.S
Lapses	0.48 ± 0.42	0.64 ± 0.45	N.S

**Table 2:** Results of backward linear regression analysis of driving questionnaires (DSI and DBQ) from independent variables (all correlations which entered the final model are presented; statistically significant results in bold letters).

Dependent Variables	N	Independent Variables							
DSI		Group (Patient/Control)	Gender	Age	BMI	ESS	FSS	AIS	StopBang
Aggression	69	-	-	-	-	-	**β = 0.027** ***p* = 0.005**	-	-
Dislike of driving	69	-	β = −0.651 *p* = 0.071	-	-	-	**β = 0.036** ***p* = 0.006**	-	-
Hazard monitoring	69	-	**β = −0.795** ***p* = 0.022**	-	**β = −0.072** ***p* = 0.032**	-	**β = 0.046** ***p* = 0.001**	-	-
Thrill seeking	69	-	**β = −0.795** ***p* = 0.023**	-	-	-	β = 0.021 *p* = 0.095	-	-
Proneness to fatigue	69	-	**β = −0.031** ***p* = 0.033**	-	-	-	-	-	-
**DBQ**									
Violations	69	-	-	**β = −0.011** ***p* = 0.021**	β = 0.021 *p* = 0.053	-	**β = 0.010** ***p* = 0.020**	-	-
Errors	69	-	-	-	**β = −0.017** ***p* = 0.016**	-	-	-	-
Lapses	69	-	-	-	-	-	-	-	-

Group: 0 control, 1 patient. Gender: 0 male, 1 female. ΒΜΙ: Body Mass Index. ESS: Epworth Sleepiness Scale. FSS: Fatigue Severity Scale. AIS: Athens Insomnia Scale. StopBang: questionnaire for obstructive sleep apnoea.

**Table 3:** Results of backward linear regression analysis between driving simulator variables and independent variables (all correlations which entered the final model are presented; statistically not significant results in italics, Bonferroni-corrected statistically significant results in bold letters).

Dependent Variables	N	Independent Variables							
		Group (Patient/Control)	Gender	Age	ΒΜΙ	ESS	FSS	AIS	StopBang
**Speed (mean)**									
Whole sample									
Speed MW	69	-	β = −7.950 *p* = 0.022	**β = −0.485** ***p* = 0.001**	-	-	-	-	-
Speed UL	69	-	β = −2.225 *p* = 0.019	**β = −0.129** ***p* = 0.001**	-	-	-	-	-
Athens sample only									
Speed UH	38	-	β = −2.313 *p* = 0.020	β = −0.100 *p* = 0.015	-	-	-	-	-
Speed RL	38	-	β = −5.892 *p* = 0.017	**β = −0.298** ***p* = 0.004**	-	-	-	-	-
SpeedRH	38	-	β = −3.989 *p* = 0.017	**β = −0.197** ***p* = 0.005**	-	-	-	-	-
**Safety margin (mean)**									
Whole sample									
HWay MW	69	-	-	-	β = −7.912 *p* = 0.010	-	-	-	β = 31.895 *p* = 0.007
THead UL	69	-	-	*β = 0.276* *p = 0.051*	-	-	-	-	-
Athens sample only									
HWay UL	38	-	-	**β = 0.928** ***p* = 0.002**	**β = −2.762** ***p* < 0.001**	**β = 2.108** ***p* = 0.003**	-	-	β = 7.090 *p* = 0.035
HWay UH	38	-	-	β = 0.537 *p* = 0.014	-	β = 1.293 *p* = 0.026	-	-	-
HWay RL	38	-	-	**β = 8.076** ***p* < 0.001**	-	-	-	-	-
HWay RH	38	-	*β = 85.764* *p = 0.094*	β = 6.002 *p* = 0.006	-	-	-	-	-
THead MW	38	-	-	β = 0.142 *p* = 0.029	-	-	-	β = 0.277 *p* = 0.043	-
THead UH	38	-	-	*β = 0.128* *p = 0.052*	-	*β = 0.309* *p = 0.079*	-	-	-
THead RL	38	β = 16.254 *p* = 0.044	-	**β = 1.443** ***p* < 0.001**	-	-	-	-	β = −8.476 *p* = 0.020
THead RH	38	-	β = 10.673 *p* = 0.041	**β = 0.730** ***p* = 0.001**	-	-	-	-	-
**Lateral Position (SD)**									
Whole sample									
RSpur MW	69	-	*β = 0.127* *p = 0.093*	**β = 0.010** ***p* = 0.002**	-	β = 0.018 *p* = 0.043	-	-	-
Athens sample only									
RSpur UL	38	-	**β = 0.526** ***p* = 0.012**	*β = −0.016* *p = 0.063*	*β = 0.043* *p = 0.051*	-	-	-	-
RSpur UH	38	**β = −0.532** ***p* = 0.006**	-	-	*β = 0.032* *p = 0.054*	-	-	**β = 0.047** ***p* = 0.004**	-
RSpur RH	38	β = 0.049 *p* = 0.038	-	-	-	-	-	-	-

Group: 0 control, 1 patient. Gender: 0 male, 1 female. ΒΜΙ: Body Mass Index. ESS: Epworth Sleepiness Scale. FSS: Fatigue Severity Scale. AIS: Athens Insomnia Scale. StopBang: questionnaire for obstructive sleep apnoea. Abbreviations of dependent variables derive from the following two components:.1. Speed (speed in kilometres per hour), HWay (distance in metres from the vehicle driving ahead), THead (time in seconds to “headway”, i.e., to collision with the vehicle driving ahead), RSpur (distance in metres from the middle of the road in metres). 2. Motorway (MW); or low (L) and high (H) traffic load conditions in urban (U) and rural (R) scenarios.

## Data Availability

The data that support the findings of this study are available on request from the corresponding author, but restrictions apply to the availability of these data, which were used under license from the First Department of Psychiatry, Eginition Hospital, National and Kapodistrian University of Athens and the collaborating centres for the current study, and so are not publicly available. Data are, however, available from the authors upon reasonable request and with permission from the First Department of Psychiatry, Eginition Hospital, National and Kapodistrian University of Athens and the collaborating centres.
